# Bilateral Spontaneous Hemothorax: A Rare Case of Primary Pleural Angiosarcoma and Literature Review †

**DOI:** 10.3390/jcm14103377

**Published:** 2025-05-12

**Authors:** Daniel Piamonti, Silvia Giannone, Letizia D’Antoni, Arianna Sanna, Nicholas Landini, Angelina Pernazza, Massimiliano Bassi, Carolina Carillo, Daniele Diso, Federico Venuta, Paolo Graziano, Pasquale Pignatelli, Lorenzo Corbetta, Matteo Bonini, Paolo Palange

**Affiliations:** 1 Department of Public Health and Infectious Diseases, Sapienza University of Rome, 00161 Rome, Italy; silvia.giannone@uniroma1.it (S.G.); letizia.dantoni@uniroma1.it (L.D.); ariannasanna1990@gmail.com (A.S.); matteo.bonini@uniroma1.it (M.B.); paolo.palange@uniroma1.it (P.P.); 2 Department of Radiological, Oncological, and Pathological Sciences, Sapienza University of Rome, 00161 Rome, Italy; nicholas.landini@uniroma1.it (N.L.); angelina.pernazza@uniorma1.it (A.P.); paolo.graziano@uniroma1.it (P.G.); 3 Department of Thoracic Surgery and Lung Transplantation, Sapienza University of Rome, 00161 Rome, Italy; massimiliano.bassi@uniroma1.it (M.B.); c.carillo@policlinicoumberto1.it (C.C.); daniele.diso@uniroma1.it (D.D.); federico.venuta@uniroma1.it (F.V.); 4 Department of Clinical, Internal, Anaesthesiological and Cardiovascular Sciences, Sapienza University of Rome, 00161 Rome, Italy; pasquale.pignatelli@uniroma1.it; 5 Unit of Interventional Pulmonology, Department of Experimental and Clinical Medicine, Careggi University Hospital, University of Florence, 50134 Florence, Italy; lorenzo.corbetta@unifi.it

**Keywords:** primary pleural angiosarcoma, pleural effusion, thoracoscopy

## Abstract

**Introduction and case report:** Angiosarcomas, rare soft tissue malignancies originating from endothelial cells, represent only 1–2% of all soft tissue sarcomas. Primary pleural angiosarcoma (PPA) is exceptionally rare, with only 43 reported cases since 1943. There are many diagnostic and therapeutic challenges due to the rarity of these tumors. We present the case of a 72-year-old man presenting with back pain, dyspnea and anemia. Conventional imaging revealed bilateral pleural effusion and a thickened parietal pleura, while contrast chest MR was able to identify pleural sites of contrast enhancement. Left chest tube placement evidenced a hemothorax, and the cytology result was negative. A thoracoscopic approach was chosen, allowing us to perform different parietal pleural biopsies. Radiological and pathological features led to the diagnosis of epithelioid PPA. Despite pleural drainage and blood transfusions, the patient died only 4 days after diagnosis. **Objectives:** To present a literature review, evaluating the disease epidemiology and the clinical, diagnostic and therapeutic features of PPA. **Methods:** We reviewed cases of PPA in the literature (1954–2024) by searching the PubMed database for the terms “pleural angiosarcoma” and “pleura + angiosarcoma”. **Results:** We found a total of 47 cases that were described between 1987 and 2024 with sufficient data to be included in our review. PPA was found to be a challenging diagnosis, found mostly in older Caucasian males. The cytology is mostly indeterminant, and an endoscopic approach is usually needed. Radical surgery is the most common treatment option, and chemotherapy and radiation therapy are also often used. However, the prognosis is poor. **Conclusions:** PPA is very rare, and complex cases such as this one showcase the importance of innovative approaches like MRI and emphasize the significance of multidisciplinary collaboration for optimal patient management. Bilateral spontaneous hemothorax, as seen in this case, is uncommon and poses additional challenges in disease management. Further research to advance the diagnostic capabilities and treatment efficacy is needed.

## 1. Introduction

Angiosarcomas are rare soft tissue malignancies derived from endothelial cells, which are part of blood or lymphatic vessels, accounting for only 1–2% of all soft tissue sarcomas. They carry a poor prognosis, also because they are characterized by a rapid growth and high metastatic potential. Also, they can arise anywhere in the body, but the most common site consists of the skin or soft tissues [1]. Rarely, they may involve the pleura [2,3]. Primary pleural angiosarcoma (PPA) is an extremely rare malignancy, with a total of 43 cases being reported in the literature since the first case was described in 1943 [2]. It is characterized by a wide variety of clinical presentations, often leading to delayed diagnosis and having poor prognosis. Also, the rarity of these tumors hinders the determination of an optimal treatment and prognosis. The description of new clinical cases of PPA can be useful in highlighting diagnostic novelties and new treatment strategies, especially in challenging cases such as our own. This case is one of the few described where PPA was complicated by bilateral spontaneous pleural bleeding, making it especially challenging in its diagnostic and therapeutic management. We also describe for the first time the use of chest MR in the diagnosis of PPA, which can be a promising tool [4,5].

## 2. Case Report

A 72-year-old Caucasian man presented to the emergency department with left-side back pain radiating anteriorly and worsening dyspnea in the past month. He had a medical history of hypertension; paraplegia secondary to spinal cord ischemia; endovascular repair of an abdominal aortic aneurysm with stent placement, complicated by type 2 endoleak; renal artery stent placement; and ureteral stent placement. Home therapy included double antiaggregant and anticoagulant therapy. The patient was a construction engineer with asbestos exposure and a former smoker of 40 pack-years. There was no history of tuberculosis or a family history of neoplastic diseases.

**Figure 1:**
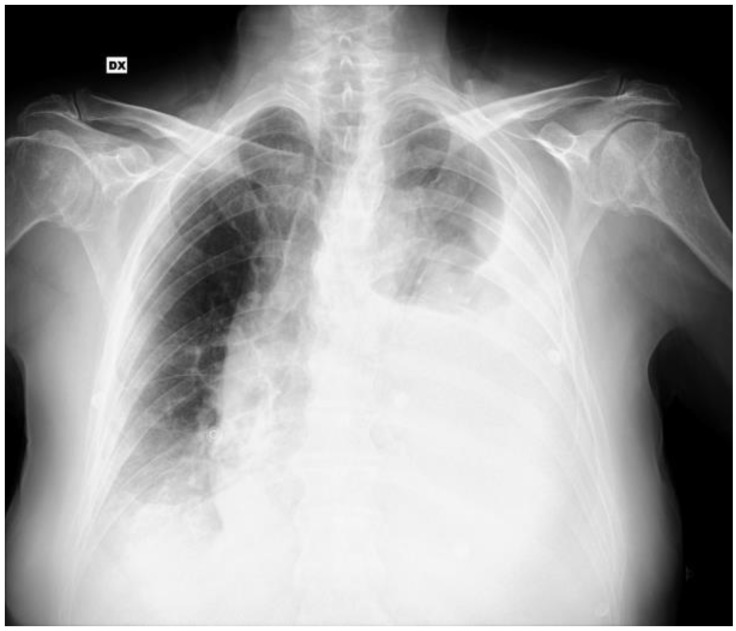
Chest radiography performed in the emergency department showing bilateral pleural effusion. DX: Right side.

On admission to the emergency department, he was eupneic, with decreased respiratory sounds bilaterally at the bases on physical examination. The laboratory tests showed normocytic normochromic anemia (hemoglobin was 6.6 g/dL), requiring blood transfusion, and a chest radiography showed bilateral pleural effusion (Figure 1).

**Figure 2:**
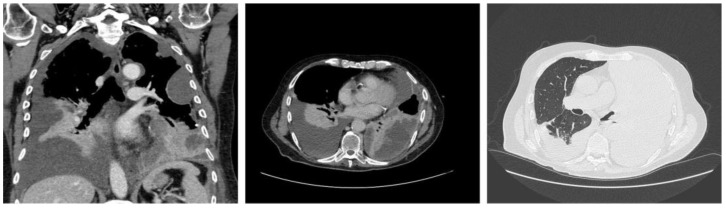
Contrast chest CT showing bilateral pleural effusion, consensual lung atelectasis, right-shifted cardiomediastinal axis, modest hyperemia, thickening of the left diaphragmatic pleural sheet and nodular thickenings of the left parietal pleura. These findings were suggestive of a malignancy involving the left parietal pleura and possibly neoplastic pleural effusion, prevalent on the left side.

A chest CT was performed, and in accordance with the hypothesis of pulmonary or pleural malignancy, contrast was also performed, highlighting bilateral pleural effusion, hyperemia and thickening of the left parietal pleura (Figure 2).

**Figure 3:**
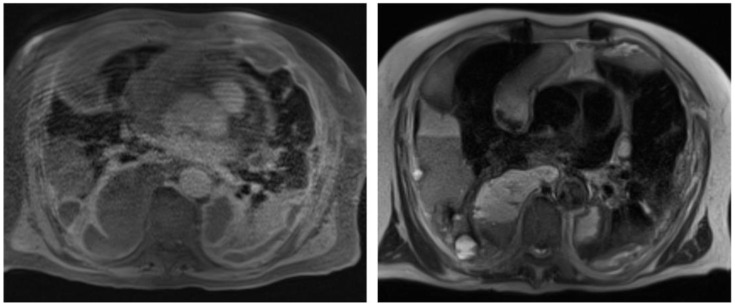
Chest contrast MR: bilateral pleural effusion with multiple saccular aspects as if due to partially bloody content. In the contrast phase, there were linear bilateral contrast enhancements of the pleura and laminar areas of restricted protonic diffusivity. These findings were consistent with pleural abnormalities, suggesting a neoplastic process, and with active bleeding in the pleural space.

Also, to better characterize the pleural abnormalities and the pleural effusion, and with the aim to obtain a better planning for a surgical approach, a contrast chest MR was performed (Figure 3).

A 12 Fr chest tube was placed in the left hemithorax, and 6 L of hematic fluid (pleural fluid Hb 8.7 g/dL and hematocrit 27.4%) was evacuated, improving dyspnea and decreasing the oxygen requirement. The cytological examination excluded the presence of neoplastic cells. Microbiological tests performed on the pleural fluid were negative.

Due to the complexity of the disease, the case was subjected to a multidisciplinary hospital team evaluation, and it was also widely discussed on “Pleural Hub”, a Facebook platform that connects various international experts on chest diseases, also for discussing complex clinical cases. A thoracoscopic approach was widely advised in order to have a direct view of the parietal pleura and to choose the most adequate sites for biopsy. Also, thoracoscopy would have enabled a visualization of the active bleeding of the pleura, and talc poudrage could have been performed if advisable. Subsequently, a left video-assisted thoracoscopy (VATS) was performed. Unilateral VATS was chosen because of the patient’s critical condition, especially due to anemia, opting for the most simple surgical approach that could lead to a diagnosis. The parietal pleura appeared thickened, diffusely hyperemic and hemorrhagic. No nodules or masses were identified. Multiple biopsies of the parietal pleura were acquired, and a talc pleurodesis was performed. A 32 Fr drainage was positioned at the end of the procedure, and during the postoperative days, we assisted to reduce the amount of pleural effusion drainage, even if the patient continued to need blood transfusions.

**Figure 4:**
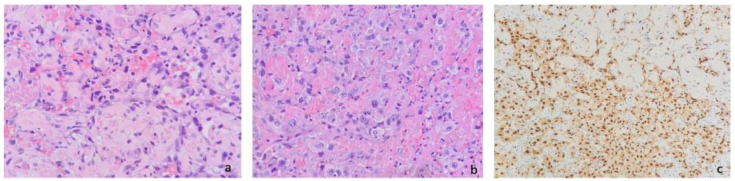
(**a**) Irregularly shaped anastomosing vascular channels, lined by atypical endothelial cells with a highly infiltrative architecture and poor demarcation (hematoxylin and eosin, magnification 10×); (**b**) solid growth pattern in poorly differentiated areas with epithelioid and pleomorphic cells (hematoxylin and eosin, magnification 20×); (**c**) in immunohistochemistry, neoplastic cells expressed ERG (endothelial marker). These findings, together with clinical and radiological data, were consistent with primary pleural epithelioid angiosarcoma.

The pathological examination enabled us to identify fibro-hematic material mixed with flaps of fibroadipose tissue with epithelioid neoplastic infiltration, partly necrotic, with the following immunophenotype: ERG+, CD31+, pancytokeratin+ (clone AE1-3), CD34−/+, D2-40−, CD15−, Ber-Ep4−, CEA−, TTF-1−, CK5/6−, calretinin−, WT1−, p16−, BAP1+ (maintained), p53+/− (wild-type), and a proliferation index (% Ki-67+) that was heterogeneously distributed and quantifiable in about 40% of neoplastic cells. The morphological and immunophenotypic findings described were consistent with a diagnosis of epithelioid angiosarcoma (Figure 4).

During hospitalization, the patient received several blood transfusions to maintain his hemoglobin level above 7 g/dL. Unfortunately, a specific oncologic treatment was never initiated due to the severe clinical condition of the patient. Exitus occurred only 4 days after the diagnosis.

## 3. Materials and Methods

We reviewed all described cases of primary pleural angiosarcoma in the recent literature (1954–2024) published in English language, using the PubMed database, by searching for the terms “pleural angiosarcoma” and “pleura + angiosarcoma”. We included in our database all cases that had enough information in order to describe the following: patients’ age, gender, main symptoms, presence of pleural effusion, outcome of pleural cytology investigation, presence of chest CT specific findings, performed diagnostic procedure and outcome, choice of treatment and time of survival. We found a total of 47 cases that included sufficient data to be analyzed and described in our review [6,7,8,9,10,11,12,13,14,15,16,17,18,19,20,21,22,23,24,25,26,27,28,29,30,31,32,33,34,35,36,37,38,39,40,41,42,43,44,45,46,47,48].

## 4. Results

All the results of our literature review are included in Table 1, which describes clinical, radiological and therapeutic aspects, summarized from a total of 47 cases that we found to be described in the literature between 1954 and 2024 [6,7,8,9,10,11,12,13,14,15,16,17,18,19,20,21,22,23,24,25,26,27,28,29,30,31,32,33,34,35,36,37,38,39,40,41,42,43,44,45,46,47,48].

## 5. Discussion

Angiosarcoma is a rare malignant tumor that accounts for only 1–2% of all soft tissue sarcomas. It arises from vascular endothelium and primarily affects the skin, breast, liver and heart [1]. PPA is an extremely rare entity [3], with only 46 cases being reported in the literature since its first description in 1943 [2]. It predominantly affects older Caucasian men. The diagnosis of PPA is challenging due to its nonspecific clinical presentation and imaging findings that may overlap with mesothelioma, lung carcinoma and metastatic adenocarcinoma. A cytological examination of pleural fluid has been negative in a significant proportion of PPA cases [3]. Thoracoscopy with biopsy is considered the most accurate diagnostic approach and should be performed promptly. A multidisciplinary evaluation is crucial for the accurate diagnosis and timely management of PPA. This involves collaboration between pulmonologists, thoracic surgeons, pathologists and oncologists. The prognosis of PPA is poor, with a median survival of approximately 4 months [2]. The presence of hemothorax is a negative prognostic factor. Radical surgery with complete resection is the primary treatment modality, while chemotherapy and radiation therapy may also be beneficial. Recently, the use of immunotherapy has been described, possibly representing a promising option for the treatment of pleural angiosarcoma [29]. However, despite these interventions, approximately 80% of patients succumb to PPA within 10 months from diagnosis [2].

The presented case highlights the diagnostic complexity of PPA, particularly in the setting of bilateral hemothorax and comorbid conditions that may preclude invasive procedures. This case highlights the limitations that are still present in diastolic pathways for PPA, especially when there is active bleeding and the patient presents with severe anemia, requiring a rapid diagnosis and prompt treatment. The patient’s imaging findings revealed bilateral pleural thickening without evident lesions, and the thoracoscopy demonstrated a thickened, hyperemic and hemorrhagic pleura without visible masses. Our case is the first ever described case that was also evaluated with MRI. Breast or cardiac angiosarcomas are frequently evaluated with MRI, as the exam is more sensitive in visualizing the lesion. Even if classic radiology techniques such as contrast chest Computed Tomography and PET/CT techniques are mostly used for the identification of pleural masses and for the planning of biopsy procedures, currently, MRI is considered the most accurate modality for characterization of a pleural lesion, local staging of the malignant tumor, and distinguishing between malignant and benign tumors [4,5]. MRI has always been underutilized in lung imaging due to two significant disadvantages: a low signal-to-noise ratio in lung tissues and susceptibility artifacts resulting from air, as well as cardiac and respiratory motion. On the other hand, this radiological technique can be useful in some cases such as the present one, allowing us to acquire a more precise localization of the tumor, when a clear lesion requiring biopsy was not seen on the CT. Bilateral involvement is a rare feature of PPA, as reported in a limited number of cases in the literature [2].

## 6. Conclusions

Primary pleural angiosarcoma is a rare and aggressive malignancy with a poor prognosis. This case is an example on how early diagnosis can be needed when the pathology is particularly aggressive, highlighting how multidisciplinary evaluation and prompt initiation of appropriate treatment are essential for optimizing patient outcomes. Our case emphasizes how further research is warranted to improve diagnostic accuracy, such as by developing new radiological approaches such as chest MR that can guide the clinician towards a rapid diagnosis, and to develop more effective treatment strategies and ultimately improve the survival of patients with this challenging disease.

## Figures and Tables

**Table 1:** Primary pleural angiosarcoma epidemiology and clinical, diagnostic and therapeutic features. The total number of cases is 47, unless otherwise specified.

**Age (Mean ± SD)**	61.8 ± 15.3 years
**Sex**	Males: 39 Females: 10
Symptoms	Dyspnea: 22 Chest pain: 15 Hemoptysis: 6 Cough: 5 No respiratory symptoms: 2 (Total described cases: 39)
Pleural effusion	No:11 (23.9%) Monolateral: 26 (56.5%) Bilateral: 9 (19.6%) Hematic effusion: 17 (48.6%) Non-hematic effusion: 18 (51.4%)
Pleural effusion cytology, when performed	Diagnostic: 3 (11.1%) Not diagnostic: 24 (88.9%) (Total described cases: 27)
CT specific findings, when performed (pleural masses or nodules)	Yes: 24 (57.1%) No: 18 (42.9%) (Total described cases: 42)
Diagnostic procedure	Cytology: 0 (0%) Thoracoscopy and pleural biopsy: 33 (71.7%) Autopsy: 7 (15.3%) CT guided biopsy: 6 (13%)
Treatment	No therapy: 11 (27.5%) Chemotherapy: 18 (45%) Radiotherapy: 3 (75%) Surgery: 7 (17.5%) Palliative care, including pleurodesis: 11 (27.5%) (Total described cases: 40)
Survival from diagnosis (mean ± SD)	20 ± 29.2 weeks

## Data Availability

The original contributions presented in this study are included in the article. Further inquiries can be directed to the corresponding author.

## Abbreviations

The following abbreviations are used in this manuscript:

PPA	primary pleural angiosarcoma
VATS	Video-Assisted Thoracoscopy
